# Preparation of Chitosan/Calcium Alginate/Bentonite Composite Hydrogel and Its Heavy Metal Ions Adsorption Properties

**DOI:** 10.3390/polym13111891

**Published:** 2021-06-07

**Authors:** Zongkun Lin, Yuru Yang, Zizhan Liang, Lei Zeng, Aiping Zhang

**Affiliations:** College of Forestry and Landscape Architecture, South China Agricultural University, Guangzhou 510642, China; zongkun_lin@163.com (Z.L.); yangyuru@stu.scau.edu.cn (Y.Y.); 20192008016@stu.scau.edu.cn (Z.L.); zenglei159753@163.com (L.Z.)

**Keywords:** chitosan, calcium alginate, bentonite, physical hydrogel, heavy metal ions adsorption

## Abstract

In order to avoid the secondary pollution of the toxic residue of chemical crosslinking agent accompanied by chemical hydrogel adsorbent and enhance the adsorption performance of physical hydrogel, chitosan/calcium alginate/bentonite (CTS/CA/BT) composite physical hydrogel was constructed. The formation mechanism and structure of the composite hydrogel were determined by FTIR, XRD and SEM. Adsorption performances of the hydrogel toward Pb^2+^, Cu^2+^ and Cd^2+^ in water under different condition as well as multi-ion competitive sorption were investigated. The adsorption processes were described with the canonical adsorption kinetics and isotherms models. With the utilization of XPS analysis and adsorption thermodynamics analysis, it was found that the adsorptions were spontaneous physico-chemical adsorptions. The results showed that the maximum adsorption capacity of the hydrogel for Pb^2+^, Cu^2+^ and Cd^2+^ reached up to 434.89, 115.30 and 102.38 mg·g^−1^, respectively, better than those of other physical hydrogels or chitosan/bentonite composite. Moreover, the composite hydrogel improved the collectability of bentonite and showed a good reusability. The modification of bentonite and the formation of hydrogel were completed simultaneously, which greatly simplifies the operation process compared with the prior similar works. These suggest that the CTS/CA/BT composite hydrogel has promising application prospects for removal of heavy metal ions from water.

## 1. Introduction

With the rapid growth of industry, significant increase of discharged heavy metal pollutants has led to numbers of environmental problems [1]. Since heavy metal ions can remain in water bodies for a long time and be enriched through the food chain, wastewater containing even a small amount of heavy metal will pose a serious threat to the human health and ecosystem [2,3]. At present, the common removal methods of heavy metal ions include chemical precipitation, coagulation, membrane filtration, electrodialysis, ion exchange and adsorption [4,5], among which, adsorption is the most widely used due to its high removal efficiency and simplicity of operator. In the adsorption process, the adsorbent is the key to achieve efficient removal of pollutants. Therefore, the development of new, high-efficiency, low-cost and easy-to-prepare adsorbents has become a research hotspot [5,6].

With a three-dimensional cross-linked network, hydrogels have been widely used in the removal of heavy metal pollutants due to their rich surface functional groups and excellent water absorption performance [7]. However, most of the currently developed hydrogel adsorbents inevitably use toxic chemical crosslinking agents for cross-linking [8,9,10]. These toxic reagents remaining in the hydrogel are difficult to be completely removed, causing secondary pollution and various adverse effects and burdens on the environment. Physical hydrogels are cross-linked through non-covalent interactions without the need for toxic chemical cross-linking agents. There have been some related studies on the adsorption of heavy metal ions by physical hydrogels [11], but their performance was mediocre. Therefore, the development of eco-friendly hydrogel adsorbents with prominent adsorption performance is of great significance to the remediation of heavy metal pollutants.

Chitosan and sodium alginate have been widely used to construct hydrogels for adsorption due to their non-toxic and environmentally friendly properties. Chitosan (CTS), one of the most abundant natural polysaccharides with rich functional groups and non-toxic, environmentally friendly properties, is an ideal candidate for heavy metal adsorption [12]. Similarly, another natural biopolymer, sodium alginate (SA), is also regarded as a prospective solution to adsorption of heavy metal [13]. However, CTS/SA hydrogel of simple structure is insufficient to provide the hydrogel with desired mechanical strength and heavy metal adsorption ability from aqueous solution [13]. In order to overcome these limitations, composite hydrogels, such as those containing clays, has been on the agenda.

The present study aimed to construct a novel and low-cost composite hydrogel adsorbent with better adsorption performance and environmental friendless in a greatly simplified operation process than the other existing works. Hereby, a chitosan/calcium alginate/bentonite (CTS/CA/BT) composite hydrogel with double-network—one is crosslinked via electrostatic interactions between chitosan and sodium alginate and the other is ionically crosslinked alginate through calcium ions—was synthesized. Double-network structure is usually proposed to improve the strength properties of hydrogel [14]. Bentonite, a kind of low-cost mineral clay, can enhance the adsorption performance of heavy metal on composite hydrogels owing to its ionic exchange capacities, surface areas and chemical/mechanical stability [15]. The combination of bentonite into double-network polymer backbone can not only improve the mechanical performances of hydrogel, but also eliminate the drawbacks of bentonite that it is difficult to recover clay particles from aqueous media after the sorption. Then the heavy metal adsorption performance and mechanism of the composite hydrogel were systematically explored utilizing Pb^2+^, Cu^2+^ and Cd^2+^ under various conditions.

## 2. Materials and Methods

### 2.1. Materials

Chitosan (deacetylation degree ≥95%, 100–200 mpa.s), sodium alginate and cadmium nitrate were purchased from Aladdin Biochemical Technology Co., Ltd. (Shanghai, China). Bentonite was obtained from Shanghai No.4 Reagent & H.V. Chemical Co., Ltd. (Shanghai, China). Acetic acid was acquired from Chinasun Specialty Products Co., Ltd. (Jiangsu, China). Sodium hydroxide, nitric acid and lead nitrate were purchased from Guangzhou Chemical Reagent Factory (Guangdong, China). Calcium carbonate (CaCO_3_) was obtained from Tianjin Kemiou Chemical Reagent Co., Ltd. (Tianjin, China). Copper nitrate was purchased from Macklin Biochemical Technology Co., Ltd. (Shanghai, China). The above reagents are of analytical grade except for the bentonite which is of chemically pure. The water used in this study is deionized water.

### 2.2. Preparation of Chitosan/Calcium Alginate/Bentonite Composite Hydrogel

Referring to and optimizing the semi-dissolution-acidification-sol-gel transition (SD-A-SGT) method reported by Zhao et al. [16], the CTS/CA/BT composite hydrogel was prepared. To be specific, with mechanical stirring, a certain amount of sodium alginate was added to distilled water to form a uniform solution. Then chitosan powder, calcium carbonate and ultrasonically dispersed bentonite were added to the sodium alginate solution successively, keeping the total mass concentration at 4%. After stirring for 2 h to form a slurry solution, poured the mixture into a Petri dish, and let it stand for 30 min to remove air bubbles. After that, the Petri dish was placed in a sealed box containing 100 mL of acetic acid for 24 h. The mixed liquid was subjected to acetic acid volatile gas and converted into a composite hydrogel. The hydrogel was rinsed with deionized water to remove acetic acid residues. A series of composite hydrogels with different mass ratios of chitosan, calcium carbonate, sodium alginate and bentonite were prepared by the same process.

### 2.3. Characterizations

The FT-IR spectra of CTS, SA, BT and freeze-dried CTS/CA/BT composite hydrogel were measured with a Thermo Scientific Nicolet iS5 spectrophotometer in the range of 4000–400 cm^−1^ at a resolution of 4 cm^−1^ for 32 scans.

The powder X-ray diffraction analysis of BT and freeze-dried CTS/CA/BT composite hydrogel were carried out with a Bruker D8 advance in a 2θ range of 5–90° using Cu Kα (λ = 0.1540 nm) radiation.

The morphology of CTS/CA/BT composite hydrogel was imaged with a Zeiss Sigma300 using the freeze-dried sample at the acceleration voltage of 3 kV.

The surface compositions of the dried CTS/CA/BT composite hydrogel before and after heavy metal ions adsorption were determined by XPS with a Thermo Scientific K-Alpha. The ray source was monochromatized Al Kα operated at 12 kV. The binding energies were calibrated with the C 1s hydrocarbon peak at 284.8 eV.

### 2.4. Heavy Metal Adsorption Experiments

To determine the heavy metal ion adsorption capacity, dried CTS/CA/BT composite hydrogel was added into the conical flasks containing heavy metal ions solution. The flasks were allowed to oscillate at a speed of 200 rpm. The concentration of heavy metal ions after the reaction was measured with an atomic absorption spectrophotometer (Shimadzu AA-7000). The Equation (1) was used to calculate the adsorption capacity.

(1) $$q_{t}=\frac{\left(C_{t}-C_{0}\right)V}{m}$$

where q_t_ (mg·g^−1^) is the adsorption capacity at time t; C_0_ (mg·L^−1^) is the initial concentration of heavy metal ions; C_t_ (mg·L^−1^) is the concentration of heavy metal ions at time t; V (mL) is the volume of heavy metal ion solution; m (mg) is the mass of the freeze-dried hydrogel.

The baseline operating conditions for the batch adsorption capacity studies were fixed as follows: dried CTS/CA/BT composite hydrogel, 10 mg; temperature, 25 °C (±1); pH, 5.0 (±0.1), adjusted with nitric acid and sodium hydroxide solution; initial concentration of metal ion solution, 500 mg·L^−1^ for Pb^2+^ and for Cu^2+^ and Cd^2+^ is 200 mg·L^−1^, respectively. Thus, for example, the effect of pH on the adsorption capacity was studied by varying the pH of metal ion solution (i.e., 1, 2, 3, 4 and 5) while using the baseline values for the other experimental parameters.

The adsorption competition and selectivity were conducted by immersing the composite hydrogel in 50 mL Pb^2+^/Cu^2+^/Cd^2+^ mixed solution with a fixed concentration (200 mg·L^−1^) for each heavy metal ion. The partition coefficient P is the ratio of the concentration of a single heavy metal ion to the sum of the concentrations of all three heavy metal ions in the sample at equilibrium, which can be calculated by Equation (2) [17]. The heavy metal with a high *p* value is the one which the composite hydrogel more likely to adsorb. The selective removal coefficient α can be calculated using Equation (3) [17]. A higher value of α means a higher adsorption selectivity.

(2) $$P=\frac{X_{i}}{\sum_{j=1}^{n}X_{j}}\left(j=1,i,\cdots n\right)$$

(3) $$\alpha =\frac{Q_{\max}/C_{x,\max}}{Q_{\min}/C_{x,\min}}$$

where the sum of the partition coefficients is equal to 1; X_i_ and X_j_ are the adsorption capacity of i and j, respectively. C_0_ for each of the three heavy metal ions is 200 mg·L^−1^. Since the initial concentrations of the three ions are the same, Q_max_ and Q_min_ are the maximum and minimum adsorption capacities of the three heavy metal ions, and C_x,max_ and C_x,max_ are the corresponding ion concentrations at equilibrium. The adsorption capacity for Pb^2+^, Cu^2+^ and Cd^2+^ ions can be calculated using Equation (1).

Interference of adsorption by K^+^ and Mg^2+^ ions was investigated by added different concentrations (i.e., 0.001, 0.01 and 0.1 mol·L^−1^) of KNO_3_ or Mg(NO_3_)_2_ into the above heavy metal ions mixed solution.

CTS/CA/BT composite hydrogels that had adsorbed Pb^2+^, Cu^2+^ and Cd^2+^ were washed with HNO_3_ solution (0.1 mol·L^−1^) for 10 h and rinsed to be neutral. After that, the composite hydrogels were immersed in the Ca^2+^ solution, then rinsed with water and freezing-dried for another cycle of adsorption. The recycling process was repeated five times.

### 2.5. Adsorption Modelling

Both linear and nonlinear adsorption models have been widely used to describe how pollutants interact with the adsorbent materials. However, the typical nonlinear adsorption models are considered to be more canonical since transformations of nonlinear equations to linear ones usually leads to parameter estimation error and distort the fit [18]. Many prior works with similar purpose did not take this into account [9,19]. Considering this, the present work utilized adsorption models in non-linear form to describe the sorption data generated from Pb^2+^, Cu^2+^ and Cd^2+^ adsorptions on CTS/CA/BT composite hydrogels more systematically. Furthermore, to identify the best-fit model, an error function was required in the optimization procedure [20]. Apart from the coefficient of determination (R^2^), the calculation of the chi-squared (χ^2^, Equation (4)) and the residual root mean square error (RMSE, Equation (5)) were also conducted, in which the smaller value indicates the better curve fitting.

(4) $$\chi^{2}=\sum_{i=1}^{m}\frac{{\left(q_{e,\exp}-q_{e,\mathrm{cal}}\right)}^{2}}{q_{e,\mathrm{cal}}}$$

(5) $$\mathrm{RMSE}=\sqrt{\frac{1}{m}\sum_{1=1}^{m}{\left(q_{e,\exp}-q_{e,\mathrm{cal}}\right)}^{2}}$$

where q_e,exp_ is the observation from the batch experiment; q_e,cal_ is the estimate from adsorption models for corresponding q_e,exp_; m is the number of observations in the experimental kinetics or isotherm.

#### 2.5.1. Adsorption Kinetics

Adsorption kinetics, playing a significant role in identifying the required equilibration time and the optimal contact time for an adsorption process, is an effective means to evaluate the adsorption efficiency of adsorbents. The measurements of adsorption kinetics should be started at an initial time of less than 2 min and ended when the adsorption process achieves true equilibrium. Therefore, the adsorption capacities of the composite hydrogel are measured at initial time of 10 s, 30 s, 1 min, 2 min, 5 min, 10 min, 15 min, 30 min, 60 min, 90 min, 120 min, 180 min, 240 min, 360 min, 480 min, 600 min, 720 min and 1440 min under the baseline operating conditions.

In this work, the pseudo-first-order (PFO) kinetic equation (Equation (6)) [21], the pseudo-second-order (PSO) kinetic equation (Equation (7)) [22], the Elovich equation (Equation (8)) [23] and the intra-particle diffusion model (Equation (9)) [24] were respectively fitted into the experimental data to investigate the kinetics behavior of CTS/CA/BT composite hydrogel for the removal of Pb^2+^, Cu^2+^ and Cd^2+^ from aqueous solution.

(6) $$q_{t}=q_{e}\left(1-e^{-k_{1}t}\right)$$

(7) $$q_{t}=\frac{q_{e}{}^{2}k_{2}t}{1+k_{2}q_{e}t}$$

where q_t_ (mg·g^−1^) is the adsorption capacity at any time t (min) and q_e_ (mg·g^−1^) is the adsorption capacity at equilibrium; k_1_ (min^−1^) and k_2_ (g·(mg·min)^−1^) is the rate constant of the PFO equation and PSO equation, respectively.

(8) $$q_{t}=\frac{1}{\beta }\ln\left(1+\alpha \beta t\right)$$

where qt (mg·g−1) is the adsorption capacity at any time t (min); α ((mg·g^−1^)·min) is the initial rate constant; β (mg·g^−1^) is the desorption constant during any one experiment.

(9) $$q_{t}{=k}_{d,i}t^{0.5}{+C}_{i}$$

where k_d,i_(g·(mg·min^−0.5^)^−1^) is the rate constant of the intra-particle diffusion model; C_i_ (mg·g^−1^) is a constant related to the thickness of the boundary layer, where a higher value of C_i_ corresponds to a greater effect on the limiting boundary layer.

#### 2.5.2. Adsorption Isotherms

Collecting adsorption isotherms is a necessary process to both understand the relation between the adsorbate concentration in aqueous solution (heavy metal ions solution) and the adsorbent (the composite hydrogel) at a constant temperature and establish adsorption programs. To arrive at valid conclusions regarding the adsorption thermodynamics, the studies should be conducted at a range of adsorbate concentrations (i.e., from 100 mg·L^−1^ to 2500 mg·L^−1^ for Pb^2+^ and from 25 mg·L^−1^ to 500 mg·L^−1^ for Cu^2+^ and Cd^2+^) and different temperatures (i.e., 298 K, 308 K and 318 K) instead of using only one initial adsorbate concentration or temperature [18].

Various models of adsorption isotherms have been applied in the literature, where the Langmuir (Equation (10)) [25] and Freundlich (Equation (12)) [26] models are the most commonly used, followed by the Dubinin–Radushkevich (Equation (13)) [27] and Redlich–Peterson (Equation (15)) [28] models. In this work, these four isotherm models were utilized to discuss the adsorption isotherm because of the practicability of their model parameters, their simplicity and their easy-interpretability.

(10) $$q_{e}=\frac{q_{m}K_{L}C_{e}}{1+K_{L}C_{e}}$$

where q_m_ (mg·g^−1^) is the maximum saturated monolayer adsorption capacity of an adsorbent; C_e_ (mg·L^−1^) is the adsorbate concentration at equilibrium; q_e_ (mg·g^−1^) is the amount of adsorption capacity at equilibrium; K_L_ (L·mg^−1^) is a constant related to the affinity between an adsorbent and adsorbate for corresponding model. The trend of adsorption can be reflected by R_L_ (Equation (7)), a constant separation factor (dimensionless) of the solid-liquid adsorption system:

(11) $$R_{L}=\frac{1}{1+K_{L}C_{0}}$$

where K_L_ is the Langmuir equilibrium constant; C_o_ (mg·L^−1^) is the initial adsorbate concentration. What is more, R_L_ = 0 means that the adsorption process is irreversible; 0 < R_L_ < 1 remarks favorable adsorption; when R_L_ > 1, it indicates unfavorable adsorption.

(12) $$q_{e}=K_{F}C_{e}{}^{n}$$

where C_e_ (mg·L^−1^) is the adsorbate concentration at equilibrium; q_e_ (mg·g^−1^) is the amount of adsorption capacity at equilibrium; K_F_ ((mg·g^−1^)·(L·mg^−1^)^n^) is the Freundlich constant; n (dimensionless) is the Freundlich intensity parameter, which indicates the magnitude of the adsorption driving force or the surface heterogeneity.

(13) $$q_{e}=q_{\mathrm{DR}}e^{-K_{\mathrm{DR}}{}^{2}}$$

(14) $$E=\frac{1}{\sqrt{2K_{\mathrm{DR}}}}$$

where q_RD_ (mg·g^−1^) is the adsorption capacity; K_RD_ (mol^2^/kJ^2^) is a constant related to the sorption energy; ɛ is the Polanyi potential; E (kJ·mol^−1^) is the mean adsorption energy which can be obtained using Equation (10).

(15) $$q_{e}=\frac{K_{\mathrm{RP}}C_{e}}{1+a_{\mathrm{RP}}C_{e}{}^{g}}$$

where a_RP_ (mg·L^−1^)^−g^ is the Redlich–Peterson constants; g (dimensionless) is an exponent whose value must lie between 0 and 1.

#### 2.5.3. Adsorption Thermodynamics

Thermodynamic study is an indispensable assistance of revealing adsorption mechanism, such as confirming whether the adsorption of heavy metal in aqueous solution is a chemical or physical process [29]. The thermodynamic parameters can be calculated in accordance with the laws of thermodynamics using Equation (16) and relationship between ΔG° and ΔH° and ΔS° is showed as Equation (17).

(16) $$\Delta G=-\mathrm{RT}\ln K_{C}$$

(17) ΔG=ΔH−TΔS

where R is the gas constant, 8.314 J·mol^−1^·K^−1^; T is the temperature in Kelvin; K_C_ (dimensionless) is the equilibrium constant at the temperature T. K_C_ values are derived from Freundlich adsorption-isotherm constants. And the linear regression coefficient (R^2^) of the Van’t Hoff equation (Equation (18)) must be high in order to guarantee the validity of K_C_.

(18) $$\ln K_{C}=\frac{-\Delta H}{R}\times \frac{1}{T}+\frac{\Delta S}{R}$$

## 3. Results and Discussion

### 3.1. Formation Mechanism of CTS/CA/BT Composite Hydrogel

**Figure 1:**
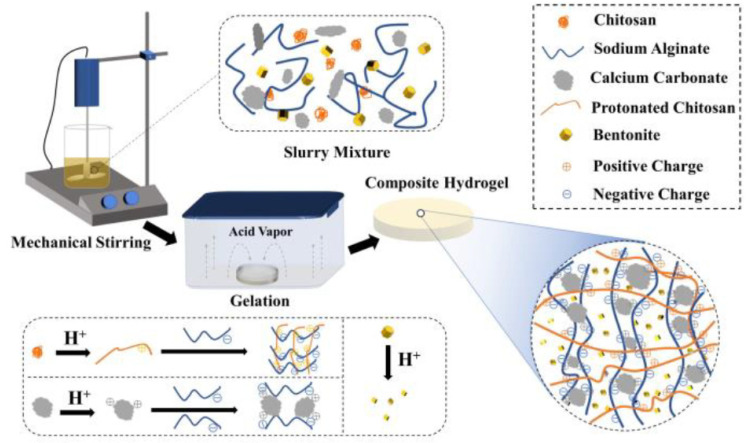
Schematic diagram of preparation process of CTS/CA/BT composite hydrogel.

The protonated chitosan can be crosslinked with sodium alginate through electrostatic interaction to form polyelectrolyte gels. The SD-A-SGT method can effectively avoid the problem of nonuniform precipitation between the cationic and anionic polyelectrolytes due to the instantaneous interaction [30,31]. As showed in Figure 1, Chitosan, calcium carbonate and bentonite powder were uniformly dispersed in the homogeneous sodium alginate solution to form a slurry mixture instead of being directly dissolved in the acetic acid solution. The slurry-like mixture was placed in a mold and exposed to an acetic acid atmosphere. As the acid gas diffused to the surface and interior of the mixture, the amino group of chitosan was slowly protonated into –NH_3_^+^ under the effect of H^+^, and crosslinked with –COO^−^ of sodium alginate through electrostatic interaction to form chitosan/sodium alginate network [32]. Calcium carbonate decomposed into calcium ions which then crosslinked with α-L-guluronic on sodium alginate to form another network [13]. Bentonite was evenly dispersed in the cross-linked network, efficiently increasing the mechanical strength of the hydrogel, forming a chitosan/calcium alginate/bentonite composite physical hydrogel. Meanwhile, bentonite was modified in an acid atmosphere [33]. The dense structure of bentonite was destroyed, and the surface turned rough, being conducive to adsorption. Moreover, the problem that bentonite is difficult to recover as an adsorbent in an aqueous medium has also been effectively solved. The synergistic effects of polymer double-network and bentonite provided an important basis for the adsorption of heavy metal on CTS/CA/BT composite hydrogel.

**Figure 2:**
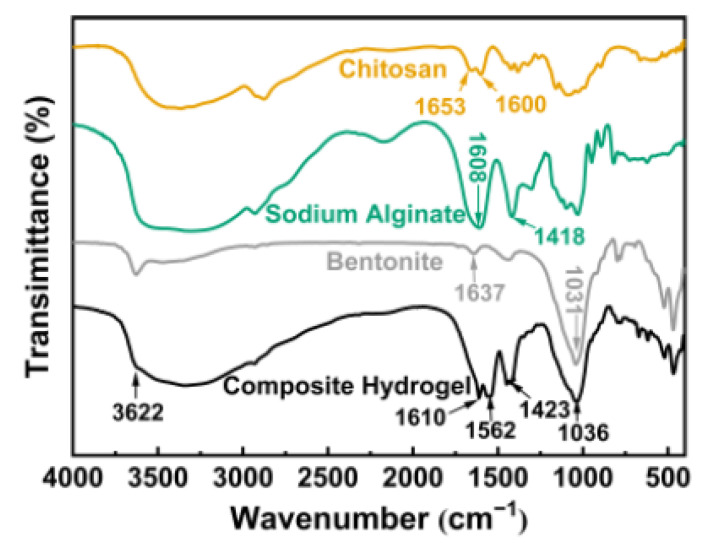
FT-IR spectra of CTS, SA, BT and CTS/CA/BT composite hydrogel.

As illustrated in Figure 2, chitosan exhibits two peaks at 1653 cm^−1^ and 1600 cm^−1^, which were caused by the tensile vibration of C=O and the bending vibration of –NH in the primary amine [34]. These two peaks were not detected in the composite hydrogel, but there was a new absorption peak at 1610 cm^−1^, indicating that the amino group on chitosan was protonated to –NH_3_^+^ [35]. It can be seen from the infrared spectrum of the composite hydrogel that the characteristic peaks of –COO^−^ in sodium alginate moved from 1608 cm^−1^ and 1418 cm^−1^ to 1562 cm^−1^ and 1423 cm^−1^, respectively, suggesting that interactions of –COO^−^ of sodium alginate with –NH_3_^+^ of chitosan and calcium ions occurred [32]. In addition, the absorption bands of bentonite at 3627 cm^−1^ and 1640 cm^−1^ were respectively caused by the bending vibration and stretching vibration of the –OH group of water used in the hydration [36]. The bound water zone of 3622 cm^−1^ and 1637 cm^−1^ in bentonite had significant changes under the effect of acid and the polymer backbone. The 1100~1000 cm^−1^ area was a Si–O band, and the peak of 1031 cm^−1^ was caused by the plane stretching of Si–O [37], indicating that the bentonite had been activated to form elongated amorphous silica [36]. These FT-IR results revealed the interactions between CTS, SA, calcium ions and bentonite.

### 3.2. Morphological Observation of CTS/CA/BT Composite Hydrogel

**Figure 3:**
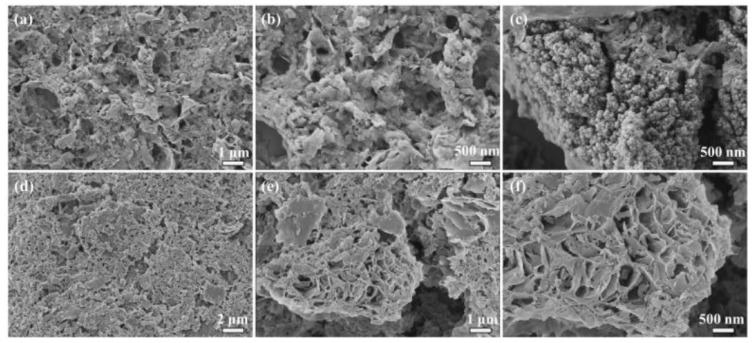
(**a**–**f**) SEM images of CTS/CA/BT composite hydrogel with different magnifications.

The SEM images of the CTS/CA/BT composite physical hydrogel under different magnifications presented in Figure 3. The figure shows that the CTS/CA/BT composite hydrogel is a heterogeneous surface and porous structure with different pore sizes. These structures are conducive to swelling which can promote the diffusion of heavy metal ions into the hydrogel [38]. The bentonite compounded with the crosslinking network, and the surface is rough with nano particles which can attract heavy metal ions.

**Figure 4:**
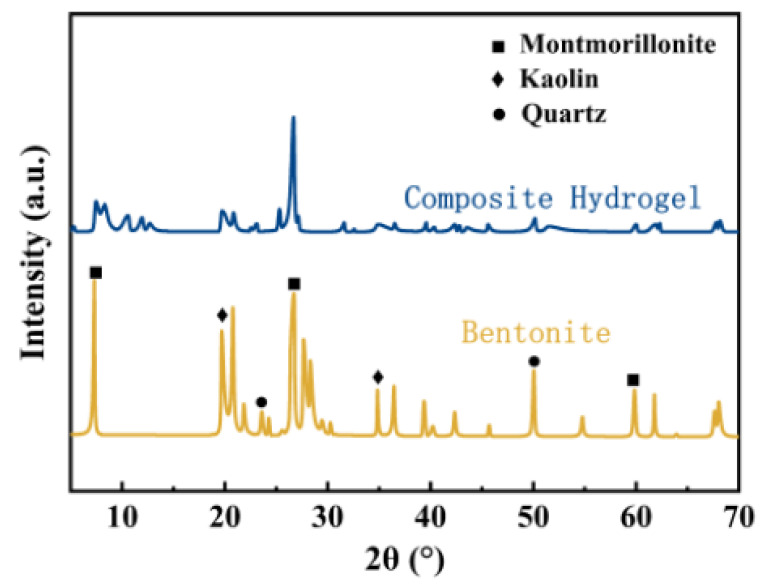
XRD patterns of bentonite and CTS/CA/BT composite hydrogel.

Coordinated with the XRD spectra in Figure 4, the diffractogram of CTS/CA/BT composite hydrogel shows the slight displacement of the CTS peaks. However, for BT, the intense peak, initially observed at d = 12.71 Å, which represents the d(001) space in BT, was obviously decreased though this shift did not occur in the spectrum of CTS/CA/BT composite hydrogel, indicating the disintegration of original compact structure to a certain extent and the formation of an exfoliated nanostructure of CTS/CA/BT composite hydrogel caused by the acid and polymer backbones [39]. Thus, the active sites were exposed, which was in favor of the adsorption of heavy metal ions by the CTS/CA/BT composite hydrogel.

### 3.3. Adsorption Performance of CTS/CA/BT Composite Hydrogel

#### 3.3.1. Adsorption Capacities of with Different Mass Ratios of Ingredient

It can be seen from Table S1 that the adsorption capacities of CTS/CA/BT composite hydrogel for Pb^2+^, Cu^2+^ and Cd^2+^ varies with the mass ratio of chitosan, sodium alginate, calcium carbonate and bentonite were determined by L_16_ (4^3^) orthogonal experiments. According to Table S1, Ki represents the sum of the corresponding heavy metal adsorption capacities when the number of hydrogels is i. From the R value, the factors affecting the heavy metal capacities are ranked as A > C > B, that is, the mass ratio of CTS to SA (greatest) and the mass ratio of CaCO_3_ to SA (smallest) influence. Meanwhile, the optimal mass ratio of ingredient for heavy metal adsorption was A_1_C_1_B_1_, namely the mass ratio of CTS to SA is 1:7; the mass ratio of CTS to SA is 1:1; mass fraction of BT is 2%. Under these conditions, the adsorption capacities of hydrogels for Pb^2+^, Cu^2+^ and Cd^2+^ reached up to 434.89, 115.30 and 102.38 mg·g^−1^, respectively, which were greater than other physical hydrogels and clay-polymer nanocomposite [32,40,41,42].

The adsorption capacities of Pb^2+^, Cu^2+^ and Cd^2+^ slightly decreased with the increase of the mass ratio of CTS to SA, which may be attributed to increasement of positive charges and reduction of negative charges brought by –NH_3_^+^ group and –COO^−^ group [43]. Similarly, due to the crosslinking of SA and Ca^2+^, higher proportion of CaCO_3_ means less metal ion binding sites, affecting the heavy metal ions adsorption capacities.

#### 3.3.2. The Influence of Solution pH on the Adsorption Performance of the Hydrogel

**Figure 5:**
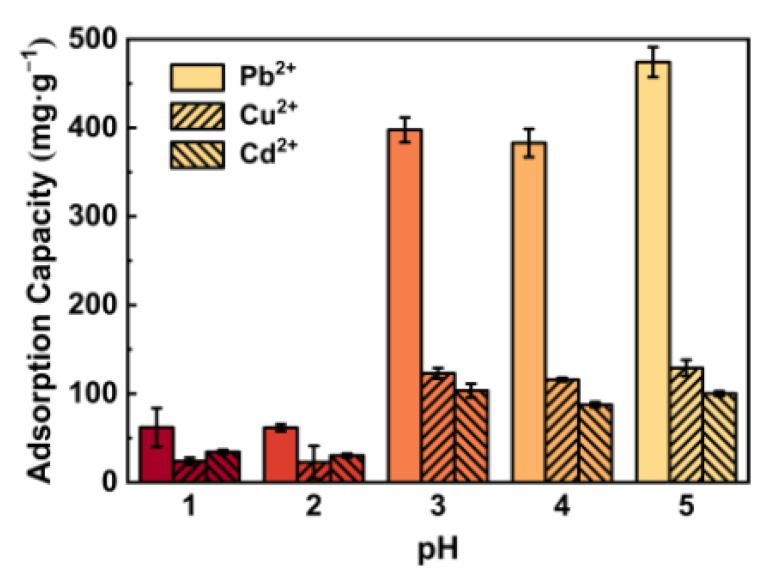
pH effect on the adsorption performance of the composite hydrogel.

Figure 5 shows the adsorption capacity of the hydrogel for various heavy metal ions at different pH. Obviously, the adsorption capacity decreased sharply at pH 1. This was due to the fact that the functional groups were protonated again under strong acid conditions, which hindered the interaction between the hydrogel and cations [44]. Under weakly acidic conditions, the adsorption capacity of the hydrogel can still be maintained at a relatively high level. The pK_a_ values of carboxylic acid groups (−COOH) are 1.7–4.7 [45]. In the aforementioned pH range, −COOH groups which were significant groups that interacted with heavy metal ions dissociated and formed negative charges. Moreover, when the pH of the heavy metal ion solution was greater than or equal to six, the solution will emerge hydroxide precipitation of metal which will affect the accuracy of the results, so experiments and discussions on this situation will not be carried out [9].

#### 3.3.3. Simultaneous Removal of Pb^2+,^ Cu^2+^ and Cd^2+^

**Figure 6:**
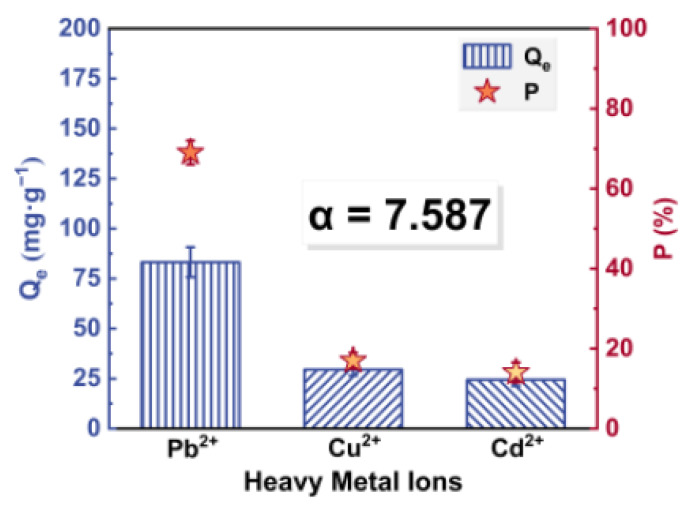
The partition coefficients and selective removal coefficient of the composite hydrogel.

Simultaneous removal of Pb^2+^, Cu^2+^ and Cd^2+^, at the same initial concentration (200 mg·L^−1^), was examined to investigate the heavy metal adsorption competition and selectivity of CTS/CA/BT composite hydrogel. As shown in Figure 6. The partition coefficients of the composite hydrogel for different heavy metal ions followed the order: Pb^2+^ (69%) > Cu^2+^ (16.96%) > Cd^2+^ (14.04%). The partition coefficient of CTS/CA/BT composite hydrogel for Pb^2+^ ions was higher than that for other ions, which may be attributed to the larger ion radius of Pb^2+^, and the negative charges on the composite hydrogel were more attractive to it. What is more, the ternary adsorption capacity was significantly lower than the single counterpart, indicating an antagonism/inhibition effect such as competition and lateral interaction between these ions on the same active sites [46].

The selective removal coefficient for CTS/CA/BT composite hydrogel was 7.587. These results demonstrate that the composite hydrogel possessed high selectivity for Pb^2+^ between Pb^2+^, Cu^2+^ and Cd^2+^. Vijayaraghavan et al. [47] indicated the electronegativity of metal cations were positively correlated with the adsorption capacity of metals on the biosorbent composite. Thus it can be seen that the adsorption on CTS/CA/BT composite hydrogel showed the selectivity sequence as Pb^2+^ > Cu^2+^ > Cd^2+^, which probably was due to the effect of the electronegativity of the metals (3.29, 2.98 and 2.71 for Pb^2+^, Cu^2+^ and Cd^2+^, respectively). That is to say, greater electronegativity leads to greater adsorption capacity. Because there is more attraction between two atoms when the electronegativity difference of nitrogen (or oxygen) and metal atom is large [48].

#### 3.3.4. Interference of Adsorption by K^+^ and Mg^2+^ Ions

**Figure 7:**
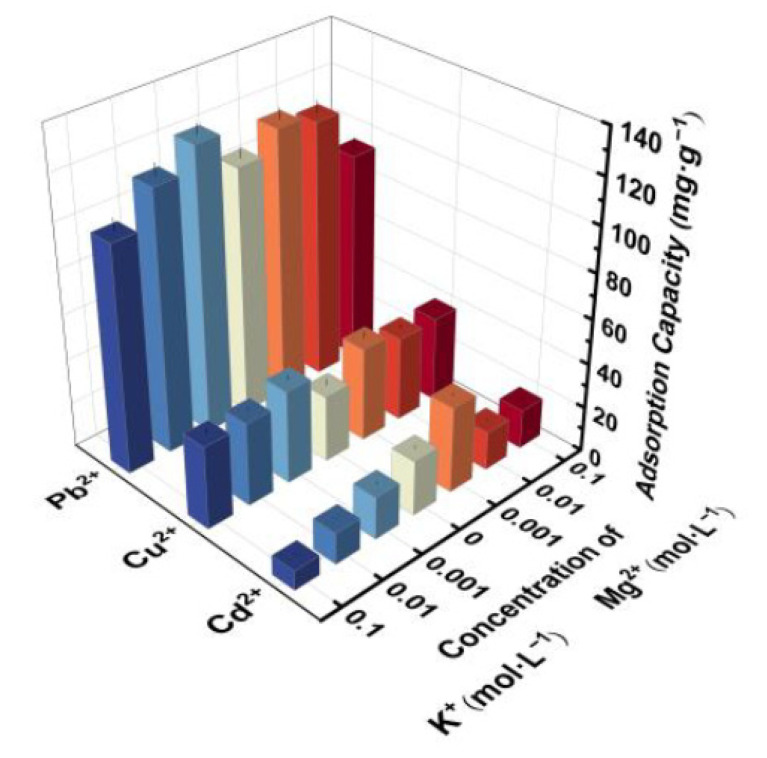
Effect of K^+^ and Mg^2+^ on the adsorption capacities of Pb^2+^, Cu^2+^ and Cd^2+^ on CTS/CA/BT composite hydrogel.

In the practical application of water treatment, coexisting ions such as K^+^ and Mg^2+^ ions are commonly seen and may affect the removal of target pollutants. Therefore, the influences of K^+^ and Mg^2+^ on the adsorption of Pb^2+,^ Cu^2+^ and Cd^2+^ by CTS/CA/BT composite hydrogel were discussed. As presented in Figure 7, the high concentration of K^+^ (0.1 mol·L^−1^) led to lower capacities of the hydrogel to adsorb Pb^2+,^ Cu^2+^ and Cd^2+^, and these may because the ion radius of K^+^ is greater than the others. However, neither K^+^ nor Mg^2+^ in a low concentration (0.01 and 0.001 mol·L^−1^) did not have negative effect on the adsorption of target ions. In contrast, a slightly increasement of the adsorption capacities occurred under these circumstances. The improvement of adsorption performance in low concentrations (0.001 M) can be explained by the addition of NO_3_^−^ (from KNO_3_ or Mg(NO_3_)_2_)which can be attached on the surface of CTS/CA/BT composite hydrogel. A high concentration of Mg^2+^ (0.1 mol·L^−1^) caused decline of the heavy metal adsorption capacities, and these may have resulted from diffusion rate of Mg^2+^ with a smaller ion radius being greater than the that of the other ions [49]. The presence of negative ions can compete for the available adsorption receptor sites of CTS/CA/BT composite hydrogel thus diminishing the electrostatic repulsion to heavy metal ions.

#### 3.3.5. Reusability of CTS/CA/BT Composite Hydrogel

**Figure 8:**
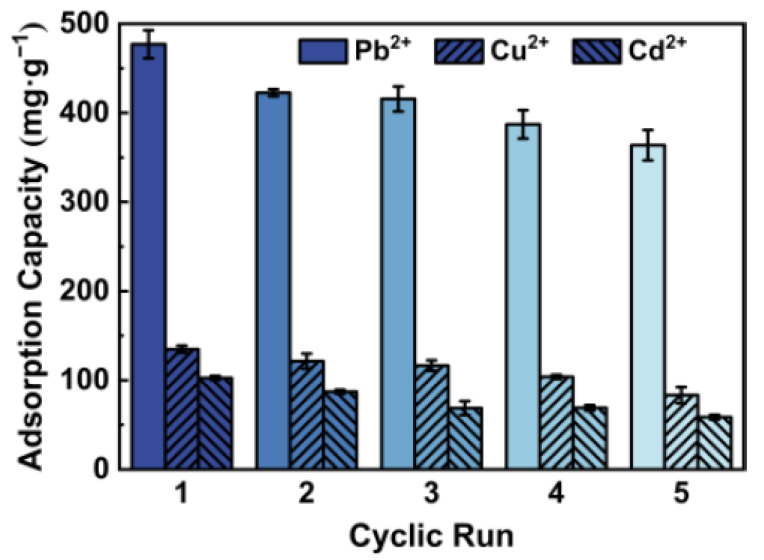
Reusability after cyclic run of CTS/CA/BT composite hydrogel.

In terms of the costs and industrial application, reusability is one of the most important indicators of whether an adsorbent is excellent or not. The reusability experiment was conducted for five continual cyclic runs. From Figure 8, after each cyclic run the adsorptions capacities of the composite hydrogel decreased due to the less Pb^2+^, Cu^2+^ and Cd^2+^ adsorption sites compared to the original state. The decrease in adsorption capacities can be attributed to looseness of the composite hydrogel caused by the loss of surface integrity of the coated polymeric material on acid treatment. The calcium ions used in the process of regeneration can minimize the impact of this, reconstructing the cross-linked network between alginate and calcium ions. In general, CTS/CA/BT composite hydrogel showed good reusability, exhibiting a promising prospect for heavy metal adsorption.

### 3.4. Adsorption Kinetics

**Figure 9:**
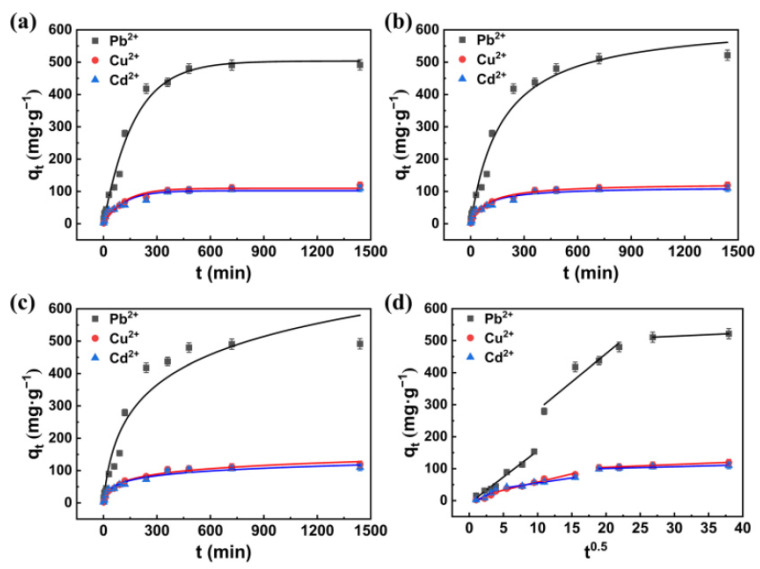
Fitting curves of adsorption kinetics. (**a**) pseudo-first-order kinetics model. (**b**) pseudo-second-order kinetics model. (**c**) Elovich model. (**d**) intra-particle diffusion model.

Figure 9 shows the kinetic fitting curve of CTS/CA/BT composite hydrogel adsorption of Pb^2+^, Cu^2+^, and Cd^2+^ and the relevant fitting parameters are shown in Table S2. By comparing the error function results of the PFO, PSO and Elovich model for Pb^2+^, it was found that the PFO and PSO kinetic models had higher correlation coefficient (R^2^) values and the PFO was able to describe the data better, as larger RMSE and chi-square values were observed in PSO model. The adsorption process of Cu^2+^ and Cd^2+^ on CTS/CA/BT composite hydrogel were quite alike. The R^2^ values of Elovich and the PSO kinetic model fitting the Cu^2+^ adsorption dates of CTS/CA/BT composite hydrogel were large, and the χ^2^ value were small, which indicated the heterogeneity of CTS/CA/BT composite hydrogel’s surface. In addition, as shown in Figure 9d, the fitting curves of intra-particle diffusion model gave multiple linear regions and did not pass through the origin, suggesting that the adsorption process was controlled by multiple steps, i.e., film diffusion (also known as external diffusion), surface diffusion or/and pore diffusion [24].

### 3.5. Adsorption Isotherms

**Figure 10:**
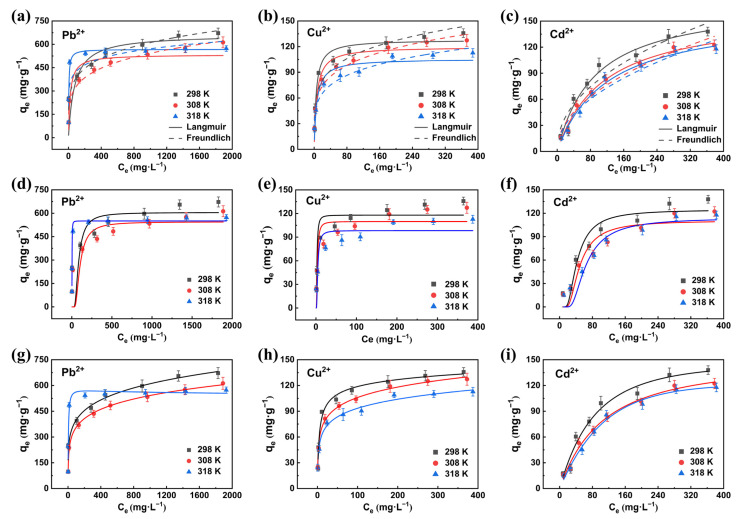
Fitting curves of adsorption isotherms. (**a**–**c**) Langmuir model and Freundlich model. (**d**–**f**) Dubinin-Radushkevich model. (**g**–**i**) Redlich-Peterson model.

The adsorption capacities at equilibrium for Pb^2+^, Cu^2+^ and Cd^2+^ increase with the increase of initial concentration because of stronger adsorption driving forces at high concentrations. As shown in Figure 10, Tables S3 and S4, at 298 K, 308 K and 318 K, the R^2^ values of Freundlich model and Redlich–Peterson model fitting the Pb^2+^, Cu^2+^ and Cd^2+^ adsorption data of CTS/CA/BT composite hydrogel are very close. However, the χ^2^ and RMSE corresponding to Freundlich model were statistically much larger than those of the Redlich–Peterson model. Therefore, the Redlich–Peterson model can describe the adsorption process more accurately than Freundlich model at all three different temperatures. These results confirmed the heterogeneity of CTS/CA/BT composite hydrogel and the extensive applicability of the Redlich–Peterson model. What is more, the model fitting results of the same heavy metal ions will varied with different temperatures, such as the adsorption process of Pb^2+^ on CTS/SA/BT composite physical hydrogel at 298, 308 and 318 K. These may be related to the energy requirement for the adsorption reaction, which may be accompanied by the change of adsorption mode, namely conversion between chemisorption and physisorption.

### 3.6. Adsorption Thermodynamics

According to the adsorption thermodynamic parameters computed and tabulated in Table 1, ΔG were negative at 298, 308 and 318 K, indicating that the adsorptions of Pb^2+^, Cu^2+^ and Cd^2+^ by the CTS/CA/BT composite physical hydrogel occurred spontaneously. As observed, the values of ΔH for Pb^2+^, Cu^2+^ and Cd^2+^ adsorption were negative, demonstrating that the adsorption were processes of exothermic. And the negative values of enthalpy revealed enhanced randomness at the CTS/CA/BT composite physical hydrogel interface during Pb^2+^, Cu^2+^ and Cd^2+^ removal process [18].

### 3.7. Adsorption Mechanism

Adsorption mechanisms cannot be directly determined based on conducting simple kinetic experiments or kinetic models fitting. Instead, the utilization of analytical techniques such as XPS, supplemented with adsorptive thermodynamic data (i.e., changes in enthalpy and entropy) and adsorption energies, are necessary in the process of assigning the adsorption mechanism.

**Figure 11:**
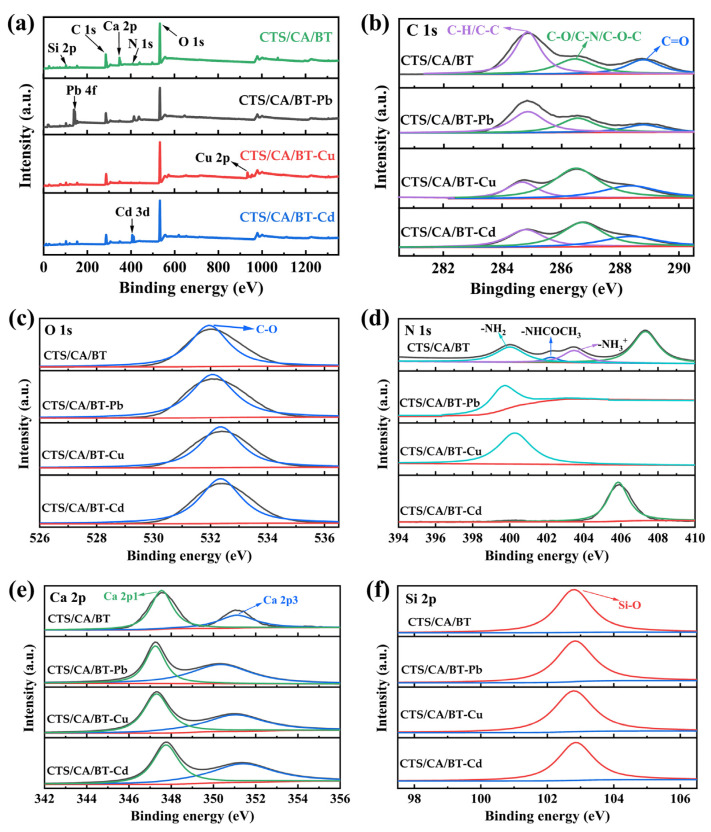
The XPS spectra of CTS/CA/BT composite hydrogel before and after adsorption: (**a**) wide-scan spectra; (**b**) C 1s spectra; (**c**) O 1s spectra; (**d**) N 1s spectra; (**e**) Ca 2p spectra; (**f**) Si 2p spectra.

The XPS spectra of CTS/CA/BT composite hydrogel before and after adsorption can be seen from Figure 11. The wide-scan spectra revealed the existence of expected signals of Pb 4f, Cu 2p and Cd 3d of CTS/CA/BT composite hydrogel after adsorption, indicating that Pb^2+^, Cu^2+^ and Cd^2+^ were absorbed on the CTS/CA/BT composite hydrogel. The appearance of these new peaks was the symbol of chemisorption. It can be seen from the (e) Ca 2p spectra that the peak intensity of Ca 2p obviously decreased, which meant that ion exchange between CTS/CA/BT composite hydrogels and Pb^2+^, Cu^2+^ and Cd^2+^ occurred [32]. The spectra of C 1s showed that the main peak intensity decreased significantly after adsorption of Cu^2+^ and Cd^2+^. The XPS spectrum of N 1s before adsorption was deconvoluted into three peaks, corresponding to the protonated ammonium ions (–NH_3_^+^), primary amine (–NH_2_) and amide (–NHCOCH_3_) in CTS/CA/BT composite hydrogels [50]. After adsorption of Pb^2+^ and Cu^2+^, the peaks of amide and ammonium ions disappeared, which can be explained by the formation of R–NH_2_–Pb^2+^/Cu^2+^ via the complexation of heavy metal ions with amide or ammonium ions. In the XPS spectra of O 1s, slight vibration in the positions of C–O was observed, indicating the existence of electrostatic interaction between heavy metal ions and CTS/CA/BT composite hydrogels [51], which is a physical effect. Therefore, the adsorption of CTS/CA/BT composite hydrogel on Pb^2+^ was also a physical adsorption. According to the fact that the adsorption occurred via both chemical and physical interactions owing to different adsorption sites in the CTS/CA/BT composite hydrogel, it can be concluded that the Pb^2+^, Cu^2+^ and Cd^2+^ adsorption of CTS/CA/BT composite hydrogel is a physico-chemical adsorption.

## 4. Conclusions

In the present work, the CTS/CA/BT composite hydrogel was prepared with low-cost and environmentally friendly ingredient—chitosan, sodium alginate, carbon calcium and bentonite—via the semi-dissolution acidification sol–gel transition method. The modification of bentonite and the formation of hydrogel were completed simultaneously, which greatly simplifies the operation process compared with the prior similar works [36,52,53]. This work was energy-efficient and eco-friendly since it was conducted under normal temperature and pressure and no toxic regents were used. The FT-IR analyses indicated that the hydrogel was composed of physically-crosslinked CTS/SA and calcium alginate self-crosslinking networks. The surface morphology of CTS/CA/BT composite hydrogel exhibits porous structure. And with the help of XRD, the formation of an exfoliated nanostructure of CTS/CA/BT composite hydrogel caused by the acid and polymer backbones was confirmed, offering numerous binding sites for the swelling and heavy metal ions adsorption. The best ratio of chitosan, sodium alginate and bentonite for Pb^2+^, Cu^2+^ and Cd^2+^ adsorption is 3:7:10. The adsorption capacity reached up to 434.89, 115.30 and 102.38 mg·g^−1^ for Pb^2+^, Cu^2+^ and Cd^2+^, respectively, which were greater than other physical hydrogels and clay-polymer nanocomposite [32,40,41,42]. In the pH range of 1~5, the optimum pH for adsorption is five, and the adsorption capacities decreased with the decrease of pH. In the ternary system of Pb^2+^, Cu^2+^ and Cd^2+^, CTS/CA/BT composite hydrogel had a high selectivity for Pb^2+^, and the introduction with low concentration of K^+^ or Mg^2+^ into the system had little effect on the adsorption properties, but high concentration of K^+^ or Mg^2+^ had slightly negative effects on the adsorption capacities of target ions. The kinetics study showed that the adsorption process of composite hydrogels can be described by pseudo-two-order kinetics or the Elovich model, and the adsorption process is controlled by multiple steps, namely, liquid film diffusion, surface diffusion and pore diffusion. The isotherm adsorption curve showed that the adsorption behavior was favorable, and it was likely to be chemical adsorption. The negative values of ΔG_0_ for the adsorptions indicate that the adsorption occurred spontaneously. XPS analysis revealed that Pb^2+^, Cu^2+^ and Cd^2+^ were adsorbed on the composite hydrogel via ion exchanges, the coordination interactions with the nitrogen atoms of CTS/CA/BT composite hydrogel and electrostatic interactions. In summary, the novel CTS/CA/BT composite hydrogel possesses brilliant application potentials in heavy metal ions removal.

## Figures and Tables

**Table 1:** Adsorption thermodynamics parameters.

	T (K)	K_C_	ΔG (kJ·mol^−1^)	ΔS (J·mol^−1^·K^−1^)	ΔH (kJ·mol^−1^)	R^2^
Pb^2+^	298	133.18	−16.35	−440.12	−147.5	0.146
	308	106.11	−11.94	−440.12	−147.5	
	318	122.75	−12.72	−423.85	−147.5	
Cu^2+^	298	35.76	−8.86	−34.84	−19.24	0.449
	308	75.83	−11.08	−26.49	−19.24	
	318	59.87	−10.82	−26.49	−19.24	
Cd^2+^	298	1.68	−1.29	−62.67	−19.97	0.996
	308	1.38	−0.82	−62.18	−19.97	
	318	1.08	−0.19	−62.18	−19.97	

## Data Availability

The data presented in this study are available on request from the first authors and corresponding author.

## Supplementary Materials

The following are available online at https://www.mdpi.com/article/10.3390/polym13111891/s1, Table S1: Orthogonal experiment of composite hydrogel’s adsorption performance, Table S2: Adsorption Kinetics Parameters, Table S3: Langmuir and Freundlich Adsorption Isotherms Parameters, Table S4: Dubinin–Radushkevich and Redlich–Peterson Adsorption Isotherms Parameters.

## References

[1] Zhou Q., Yang N., Li Y., Ren B., Ding X., Bian H., Yao X. (2020). Total concentrations and sources of heavy metal pollution in global river and lake water bodies from 1972 to 2017. Glob. Ecol. Conserv..

[2] Akpor O.B. (2014). Heavy Metal Pollutants in Wastewater Effluents: Sources, Effects and Remediation. Adv. Biosci. Bioeng..

[3] Man Y., Wang B., Wang J., Slaný M., Yan H., Li P., El-Naggar A., Shaheen S.M., Rinklebe J., Feng X. (2021). Use of biochar to reduce mercury accumulation in Oryza sativa L: A trial for sustainable management of historically polluted farmlands. Environ. Int..

[4] Crini G., Lichtfouse E. (2019). Advantages and disadvantages of techniques used for wastewater treatment. Environ. Chem. Lett..

[5] Wang Q., Shaheen S.M., Jiang Y., Li R., Slaný M., Abdelrahman H., Kwon E., Bolan N., Rinklebe J., Zhang Z. (2021). Fe/Mn- and P-modified drinking water treatment residuals reduced Cu and Pb phytoavailability and uptake in a mining soil. J. Hazard. Mater..

[6] Burakov A.E., Galunin E.V., Burakova I.V., Kucherova A.E., Agarwal S., Tkachev A.G., Gupta V.K. (2018). Adsorption of heavy metals on conventional and nanostructured materials for wastewater treatment purposes: A review. Ecotoxicol. Environ. Saf..

[7] Ahmed E.M. (2015). Hydrogel: Preparation, characterization, and applications: A review. J. Adv. Res..

[8] Chen X., Li P., Kang Y., Zeng X., Xie Y., Zhang Y., Wang Y., Xie T. (2019). Preparation of temperature-sensitive Xanthan/NIPA hydrogel using citric acid as crosslinking agent for bisphenol A adsorption. Carbohydr. Polym..

[9] Zhao B., Jiang H., Lin Z., Xu S., Xie J., Zhang A. (2019). Preparation of acrylamide/acrylic acid cellulose hydrogels for the adsorption of heavy metal ions. Carbohydr. Polym..

[10] Sharifzadeh G., Hezaveh H., Muhamad I.I., Hashim S., Khairuddin N. (2020). Montmorillonite-based polyacrylamide hydrogel rings for controlled vaginal drug delivery. Mater. Sci. Eng. C.

[11] Yu C., Tang X., Liu S., Yang Y., Shen X., Gao C. (2018). Laponite crosslinked starch/polyvinyl alcohol hydrogels by freezing/thawing process and studying their cadmium ion absorption. Int. J. Biol. Macromol..

[12] Rinaudo M. (2006). Chitin and chitosan: Properties and applications. Prog. Polym. Sci..

[13] Wang B., Wan Y., Zheng Y., Lee X., Liu T., Yu Z., Huang J., Ok Y.S., Chen J., Gao B. (2018). Alginate-based composites for environmental applications: A critical review. Crit. Rev. Environ. Sci. Technol..

[14] Bi S., Wang P., Hu S., Li S., Pang J., Zhou Z., Sun G., Huang L., Cheng X., Xing S. (2019). Construction of physical-crosslink chitosan/PVA double-network hydrogel with surface mineralization for bone repair. Carbohydr. Polym..

[15] Adeyemo A.A., Adeoye I.O., Bello O.S. (2017). Adsorption of dyes using different types of clay: A review. Appl. Water Sci..

[16] Zhao J., Chen Y., Yao Y., Tong Z.-R., Li P.-W., Yang Z.-M., Jin S.-H. (2018). Preparation of the polyelectrolyte complex hydrogel of biopolymers via a semi-dissolution acidification sol-gel transition method and its application in solid-state supercapacitors. J. Power Sources.

[17] Liu M., Liu Y., Shen J., Zhang S., Liu X., Chen X., Ma Y., Ren S., Fang G., Li S. (2020). Simultaneous removal of Pb^2+^, Cu^2+^ and Cd^2+^ ions from wastewater using hierarchical porous polyacrylic acid grafted with lignin. J. Hazard. Mater..

[18] Tran H.N., You S.-J., Hosseini-Bandegharaei A., Chao H.-P. (2017). Mistakes and inconsistencies regarding adsorption of contaminants from aqueous solutions: A critical review. Water Res..

[19] Ghosal P.S., Gupta A.K. (2017). Determination of thermodynamic parameters from Langmuir isotherm constant-revisited. J. Mol. Liq..

[20] Vijayaraghavan K., Padmesh T., Palanivelu K., Velan M. (2006). Biosorption of nickel(II) ions onto Sargassum wightii: Application of two-parameter and three-parameter isotherm models. J. Hazard. Mater..

[21] Lagergren S. (1898). About the theory of so-called adsorption of soluble substances. Sven Vetensk. Handingarl.

[22] Blanchard G., Maunaye M., Martin G. (1984). Removal of heavy metals from waters by means of natural zeolites. Water Res..

[23] McLintock I.S. (1967). The Elovich Equation in Chemisorption Kinetics. Nat. Cell Biol..

[24] Weber W.J., Morris J.C. (1963). Kinetics of adsorption on carbon from solution. J. Sanit. Eng. Div..

[25] Langmuir I. (1918). The adsorption of gases on plane surfaces of glass, mica and platinum. J. Am. Chem. Soc..

[26] Freundlich H.M.F., Chem Z.P. (1906). Über die adsorption in lösungen. Z. Phys. Chem..

[27] Dubinin M.M., Radushkevich L.V. (1947). Equation of the Characteristic Curve of Activated Charcoal. Proc. USSR Acad. Sci..

[28] Redlich O., Peterson D.L. (1959). A Useful Adsorption Isotherm. J. Phys. Chem..

[29] Tran H.N., You S.-J., Chao H.-P. (2016). Thermodynamic parameters of cadmium adsorption onto orange peel calculated from various methods: A comparison study. J. Environ. Chem. Eng..

[30] Luo F., Sun T.L., Nakajima T., Kurokawa T., Zhao Y., Sato K., Bin Ihsan A., Li X., Guo H., Gong J.P. (2015). Oppositely Charged Polyelectrolytes Form Tough, Self-Healing, and Rebuildable Hydrogels. Adv. Mater..

[31] Schoubben A., Blasi P., Giovagnoli S., Rossi C., Ricci M. (2010). Development of a scalable procedure for fine calcium alginate particle preparation. Chem. Eng. J..

[32] Tang S., Yang J., Lin L., Peng K., Chen Y., Jin S., Yao W. (2020). Construction of physically crosslinked chitosan/sodium alginate/calcium ion double-network hydrogel and its application to heavy metal ions removal. Chem. Eng. J..

[33] Pawar R.R., Ingole P.G., Lee S.-M. (2020). Use of activated bentonite-alginate composite beads for efficient removal of toxic Cu^2+^ and Pb^2+^ ions from aquatic environment. Int. J. Biol. Macromol..

[34] Su Z., Han Q., Zhang F., Meng X., Liu B. (2020). Preparation, characterization and antibacterial properties of 6-deoxy-6-arginine modified chitosan. Carbohydr. Polym..

[35] Fernandez J.G., Ingber N.E. (2011). Unexpected Strength and Toughness in Chitosan-Fibroin Laminates Inspired by Insect Cuticle. Adv. Mater..

[36] Pawar R.R., Bajaj H.C., Lee S.-M. (2016). Activated bentonite as a low-cost adsorbent for the removal of Cu(II) and Pb(II) from aqueous solutions: Batch and column studies. J. Ind. Eng. Chem..

[37] Slaný M., Jankovič Ľ., Madejová J. (2019). Structural characterization of organo-montmorillonites prepared from a series of primary alkylamines salts: Mid-IR and near-IR study. Appl. Clay Sci..

[38] Chang Z., Chen Y., Tang S., Yang J., Chen Y., Chen S., Li P., Yang Z. (2020). Construction of chitosan/polyacrylate/graphene oxide composite physical hydrogel by semi-dissolution/acidification/sol-gel transition method and its simultaneous cationic and anionic dye adsorption properties. Carbohydr. Polym..

[39] Dotto G.L., Rodrigues F., Tanabe E., Fröhlich R., Bertuol D., Martins T.R., Foletto E. (2016). Development of chitosan/bentonite hybrid composite to remove hazardous anionic and cationic dyes from colored effluents. J. Environ. Chem. Eng..

[40] Yang S.C., Liao Y., Karthikeyan K.G., Pan X.J. (2021). Mesoporous cellulose-chitosan composite hydrogel fabricated via the co-dissolution-regeneration process as biosorbent of heavy metals. Environ. Pollut..

[41] Vieira R.M., Vilela P.B., Becegato V.A., Paulino A.T. (2018). Chitosan-based hydrogel and chitosan/acid-activated montmorillonite composite hydrogel for the adsorption and removal of Pb^2+^ and Ni^2+^ ions accommodated in aqueous solutions. J. Environ. Chem. Eng..

[42] Ma J., Luo J., Liu Y., Wei Y., Cai T., Yu X., Liu H., Liu C., Crittenden J.C. (2018). Pb(ii), Cu(ii) and Cd(ii) removal using a humic substance-based double network hydrogel in individual and multicomponent systems. J. Mater. Chem. A.

[43] Jiang W., Chen X., Pan B., Zhang Q., Teng L., Chen Y., Liu L. (2014). Spherical polystyrene-supported chitosan thin film of fast kinetics and high capacity for copper removal. J. Hazard. Mater..

[44] Baghbadorani N.B., Behzad T., Etesami N., Heidarian P. (2019). Removal of Cu^2+^ ions by cellulose nanofibers-assisted starch-g-poly(acrylic acid) superadsorbent hydrogels. Compos. Part B Eng..

[45] Volesky B. (2007). Biosorption and me. Water Res..

[46] Sellaoui L., Soetaredjo F.E., Ismadji S., Bonilla-Petriciolet A., Belver C., Bedia J., Ben Lamine A., Erto A. (2018). Insights on the statistical physics modeling of the adsorption of Cd^2+^ and Pb^2+^ ions on bentonite-chitosan composite in single and binary systems. Chem. Eng. J..

[47] Vijayaraghavan K., Teo T.T., Balasubramanian R., Joshi U.M. (2009). Application of Sargassum biomass to remove heavy metal ions from synthetic multi-metal solutions and urban storm water runoff. J. Hazard. Mater..

[48] Hu C., Zhu P., Cai M., Hu H., Fu Q. (2017). Comparative adsorption of Pb(II), Cu(II) and Cd(II) on chitosan saturated montmorillonite: Kinetic, thermodynamic and equilibrium studies. Appl. Clay Sci..

[49] Wu J., Wang T., Wang J., Zhang Y., Pan W.-P. (2021). A novel modified method for the efficient removal of Pb and Cd from wastewater by biochar: Enhanced the ion exchange and precipitation capacity. Sci. Total. Environ..

[50] Wu S., Yan K., Zhao Y., Tsai C.-C., Shen J., Bentley W.E., Chen Y., Deng H., Du Y., Payne G.F. (2018). Electrical Writing onto a Dynamically Responsive Polysaccharide Medium: Patterning Structure and Function into a Reconfigurable Medium. Adv. Funct. Mater..

[51] Huang Z., Huang Z., Feng L., Luo X., Wu P., Cui L., Mao X. (2018). Modified cellulose by polyethyleneimine and ethylenediamine with induced Cu(II) and Pb(II) adsorption potentialities. Carbohydr. Polym..

[52] Sakr M.A., Mohamed M.G., Wu R., Shin S.R., Kim D., Kim K., Siddiqua S. (2020). Development of bentonite-gelatin nanocomposite hybrid hydrogels for tissue engineering. Appl. Clay Sci..

[53] Chkirida S., Zari N., Achour R., Hassoune H., Lachehab A., Qaiss A.E.K., Bouhfid R. (2021). Highly synergic adsorption/photocatalytic efficiency of Alginate/Bentonite impregnated TiO_2_ beads for wastewater treatment. J. Photochem. Photobiol. A: Chem..

