# Supplementary material for: Preparation of Chitosan/Calcium Alginate/Bentonite Composite Hydrogel and Its Heavy Metal Ions Adsorption Properties
Source: Polymers (Basel). 2021 Jun 7;13(11):1891. doi: 10.3390/polym13111891 (PMC8201196; doi:10.3390/polym13111891)
Supplement: Supplementary file 1 [file polymers-13-01891-s001.zip › polymers-1229837-supplementary.pdf]

Supplementary Information

Preparation of Chitosan/Calcium Alginate/Bentonite Composite hydrogel and its heavy metal ions adsorption properties

Zongkun Lin †, Yuru Yang †, Zizhan Liang, Lei Zeng and Aiping Zhang *

College of Forestry and Landscape Architecture, South China Agricultural University, Guangzhou 510642, PR China; linzongkun@qq.com(Z.Lin); 3247315952@qq.com(Y.Yang);

* Correspondence: aiping@scau.edu.cn; Tel.: +86-159-1311-8266

† Co-first author, these authors contributed equally to this work.

Table S1. Orthogonal experiment of composite hydrogel's adsorption performance

Number of Hydrogels		*A* m_{CTS} ∶ m_{SA}	*B* m_{CaCO3} ∶ m_{SA}	C Mass fraction of BT (wt.%)	$Q_{Pb^{2+}}$	$Q_{Cu^{2+}}$	$Q_{Cd^{2+}}$
1		a (1 ∶ 7)	a (1 ∶ 1)	a (2)	335.36	115.30	102.38
2		a	b (2 ∶ 1)	b (4)	268.64	91.68	82.45
3		a	c (3 ∶ 1)	c (6)	286.16	75.89	74.05
4		a	d (4 ∶ 1)	d (8)	434.89	73.62	48.2
5		b (2 ∶ 6)	a	b	212.77	62.68	40.35
6		b	b	a	389.03	94.93	72.55
7		b	c	d	201.04	69.63	55.78
8		b	d	c	395.04	71.21	49.63
9		c (3 ∶ 5)	a	c	269.01	72.53	38.33
10		c	b	d	70.24	64.94	35.15
11		c	c	a	362.42	91.41	46.87
12		c	d	b	113.09	61.48	55.88
13		d (4 ∶ 4)	a	d	39.70	84.41	5.32
14		d	b	c	30.32	60.37	34.41
15		d	c	b	47.73	70.09	5.13
16		d	d	a	340.05	85.71	18.25
	Pb^{2+}	331.26	232.56	356.72			
$\overline{K}_a$	Cu^{2+}	89.12	83.73	96.84			
	Cd^{2+}	76.77	46.60	60.01			
	Pb^{2+}	299.47	189.56	160.56			
$\overline{K}_b$	Cu^{2+}	74.61	77.98	71.48			
	Cd^{2+}	54.58	56.14	45.95			
	Pb^{2+}	203.69	224.34	245.13			
$\overline{K}_c$	Cu^{2+}	72.59	76.76	70.00			
	Cd^{2+}	44.06	45.46	49.11			
	Pb^{2+}	114.45	320.77	186.47			
$\overline{K}_d$	Cu^{2+}	75.15	73.01	73.15			
	Cd^{2+}	15.78	42.99	36.11			
	Pb^{2+}	216.81	131.21	196.16			
R	Cu^{2+}	16.53	10.73	26.84			
	Cd^{2+}	60.99	13.15	23.90			

Influence degree	A>C>B	C>A>B	A>C>B
Optimization		$A_1C_1B_1$	

Table S2. Adsorption Kinetics Parameters

Models	Parameters	Values		
		Pb^{2+}	Cu^{2+}	Cd^{2+}
Pseudo-first-order	K_1	0.00541	0.00863	0.0081
Model	$q_{e,1}$	503.539	109.392	102.093
	R^2	0.984	0.956	0.901
	RMSE	23.14	7.921	10.756
	χ^2	25.156	8.611	11.694
Pseudo-second-order	K_2	9.06×10^{-6}	8.76×10^{-5}	1.12×10^{-4}
Model	$q_{e,2}$	629.566	124.417	113.503
	R^2	0.97425	0.98	0.941
	RMSE	30.207	5.261	8.281
	χ^2	32.838	6.14	9
Elovich Model	α	5.641	2.82525	3.560
	β	0.007	0.04	0.0468
	R^2	0.932	0.984	0.966
	RMSE	47.612	4.74	6.249
	χ^2	51.76	5.153	6.794
Intra-particle Diffusion	$K_{d,1}$	26.27	4.069	11.317
Model	C_1	-56.36	-1.859	-11.781
	R^2	0.9324	1	0.903
	$K_{d,2}$	16.466	5.069	3.212
	C_2	-8.484	7.422	22.9
	R^2	0.972	0.967	0.959
	$K_{d,3}$	0.979	0.845	0.556
	C_3	484.535	87.495	89.46
	R^2	1	0.996	0.873

Table S3. Langmuir and Freundlich Adsorption Isotherms Parameters

	T (K)	Langmuir q_m	K_L	R_L	R^2	RMSE	χ^2	Freundlich K_F	n	R^2	RMSE	χ^2
Pb^{2+}	298	662.37	0.013	0.43~0.03	0.74	90.7	104.72	157.54	0.2	0.95	37.58	43.4
	308	533.81	0.046	0.18~0.0086	0.86	57.54	66.44	125.26	0.21	0.98	22.59	26.08
	318	567.80	0.24	0.041~0.0017	0.95	36.48	42.11	241.37	0.13	0.7	85.77	99.04
Cu^{2+}	298	128.05	0.2	0.14~0.00816	0.95	7.61	8.78	44.082	0.20	0.88	12.02	13.88
	308	119.73	0.16	0.201~0.0124	0.92	9.47	10.92	40.044	0.2	0.97	6.041	6.98
	318	105.72	0.16	0.2~0.012	0.904	8.6	9.93	35.684	0.20	0.96	5.59	6.57
Cd^{2+}	298	128.04	0.2	0.17~0.0098	0.962	7.61	8.78	44.082	0.034	0.88	12.02	13.86
	308	119.73	0.16	0.2~0.012	0.986	9.46	10.91	40.044	0.019	0.97	6.041	6.93
	318	105.72	0.16	0.2~0.012	0.985	3.04	9.9	35.684	0.021	0.96	5.69	6.56

Table S4. Dubinin-Radushkevich and Redlich-Peterson Adsorption Isotherms Parameters

	T (K)	Dubinin-Radushkevich q_{DR} ($mg\cdot g^{-1}$)	K_{DR} (mol^2/kJ^2)	E ($kJ\cdot mol^{-1}$)	R^2	RMSE	χ^2	Redlich-Peterson K_{RP}	a_{RP} ($(mg\cdot L^{-1})^{-g}$)	g	R^2	RMSE	χ^2
Pb^{2+}	298	604.77	8.92×10^{-2}	23.67	0.63	122.51	106.1	733.18	4.23	0.82	0.99	9.85	12.45
	308	544.94	1.27×10^{-3}	19.87	0.58	114.77	99.39	106.11	0.63	0.83	0.99	38.93	46.71
	318	550.52	1.31×10^{-6}	618.38	0.87	31.84	27.57	122.75	0.19	1.02	0.94	35.28	44.63
Cu^{2+}	298	117.95	9.73×10^{-7}	716.78	0.89	14.6	12.64	35.76	0.41	0.93	0.97	5.62	7.12
	308	109.78	1.79×10^{-6}	527.95	0.79	18.56	16.07	75.83	1.39	0.85	0.99	2.34	2.97
	318	98.26	2.67×10^{-6}	433.07	0.78	16.4	14.21	59.87	1.22	0.86	0.99	3.085	3.9
Cd^{2+}	298	124.48	2.04×10^{-4}	49.56	0.91	13.95	12.09	1.68	0.0046	1.13	0.96	7.06	9.62
	308	110.49	3.02×10^{-4}	40.68	0.84	14.52	12.58	1.38	0.0062	1.05	0.98	4.09	5.18
	318	114	4.57×10^{-4}	33.07	0.9	12.83	11.11	1.07	0.0013	1.27	0.99	3.56	4.5
